# Extrapulmonary hyalinizing granuloma: a rare case with intra-articular and tenosynovial involvement

**DOI:** 10.1007/s00256-024-04762-9

**Published:** 2024-07-24

**Authors:** Mohanad Alhumayed, Ryan P. Austin, Eric Y. Chang

**Affiliations:** 1 Department of Radiology, University of California San Diego, San Diego, CA 92103 USA; 2 Department of Pathology, Naval Hospital Camp Pendleton, Oceanside, CA 92055 USA; 3 Radiology Service, VA San Diego Healthcare System, San Diego, CA 92161 USA

**Keywords:** Extrapulmonary hyalinizing granuloma, Synovial masses, Synovitis, Rheumatological imaging, Magnetic resonance imaging, Wrist joint

## Abstract

Extrapulmonary hyalinizing granuloma (EPHG) is a notably rare condition, representing an exaggerated chronic immune response to antigenic stimuli. This report presents the first documented case of intra-articular and tenosynovial EPHG with radiological evaluation and pathological confirmation in a 60-year-old man presenting with wrist pain and swelling. Imaging findings were relatively symmetric with marked distension of the distal radioulnar joints and extensor tendon sheaths with masses and nodules of various sizes surrounded by synovitis and accompanied by bony erosions. On US, the masses were heterogeneous but mostly hypo- to iso-echoic compared to muscle and relatively hypovascular. On MRI, compared to muscle, the nodules exhibited iso-intense signal on T1-weighted images, iso- to mildly hyper-intense signal on T2-weighted fat-suppressed images, and minimal enhancement on post-contrast images. The diagnosis of EPHG was revealed through biopsy and pathologic examination with glucocorticoids being effective in treatment.

## Introduction

Pulmonary hyalinizing granuloma (PHG) is a rare lung disease first described by Engleman et al. in 1977 [1]. Histologically characterized by concentric hyaline lamellae often surrounded by perivascular collections of plasma cells and lymphocytes, PHG is frequently associated with conditions like mediastinal and retroperitoneal fibrosis, autoimmune disorders, hematologic abnormalities, thromboembolic events, and infectious diseases [1, 2]. The occurrence of extrapulmonary hyalinizing granuloma (EPHG) is exceedingly rare, with documented instances in locations including the brain, orbit, neck, chest wall, mediastinum, abdominal cavity, and lymphatic system [3–7]. The etiology of PHG, and by extension EPHG, remains unknown. Numerous reports suggest that patients with PHG often exhibit autoimmune phenomena, leading to the hypothesis that PHG may represent an exaggerated chronic immune response [1, 8]. This theory is likely applicable to EPHG as well, given their shared pathology. Definitive diagnosis for both conditions is established through histopathological examination of the lesions [1, 9]. To our knowledge, this is the first documented case of isolated EPHG involving the musculoskeletal system, marking an exceptionally rare manifestation of this condition.

## Case report

A 60-year-old man with a significant medical history of chronic lymphocytic leukemia (CLL), immune thrombocytopenic purpura, hypertension, and hyperlipidemia presented for evaluation of progressively worsening bilateral wrist pain and swelling. Symptoms had worsened over six months and were now impacting his daily function. Physical exam demonstrated multiple mildly tender masses about the wrists, and imaging was ordered to evaluate for potential malignancy given the history of leukemia. Laboratory tests showed an elevated erythrocyte sedimentation rate at 57 mm/h (normal < 20 mm/h) and C-reactive protein levels at 4.23 mg/dL (normal < 0.8 mg/dL). Antinuclear antibody and antinuclear ribonucleoprotein tests were positive, but are nonspecific and seen with many rheumatologic and non-rheumatologic conditions. Rheumatoid factor and anti-cyclic citrullinated peptide antibody tests were negative. 18F-fluorodeoxyglucose positron emission tomography-computed tomography (18F-FDG PET-CT) was obtained from the skull through the thighs, showing multiple mildly enlarged, non-hypermetabolic lymph nodes throughout the body, consistent with the patient’s history of CLL. Nonspecific hypermetabolic activity was present about both wrists. No pulmonary abnormalities were seen.

**Fig. 1:**
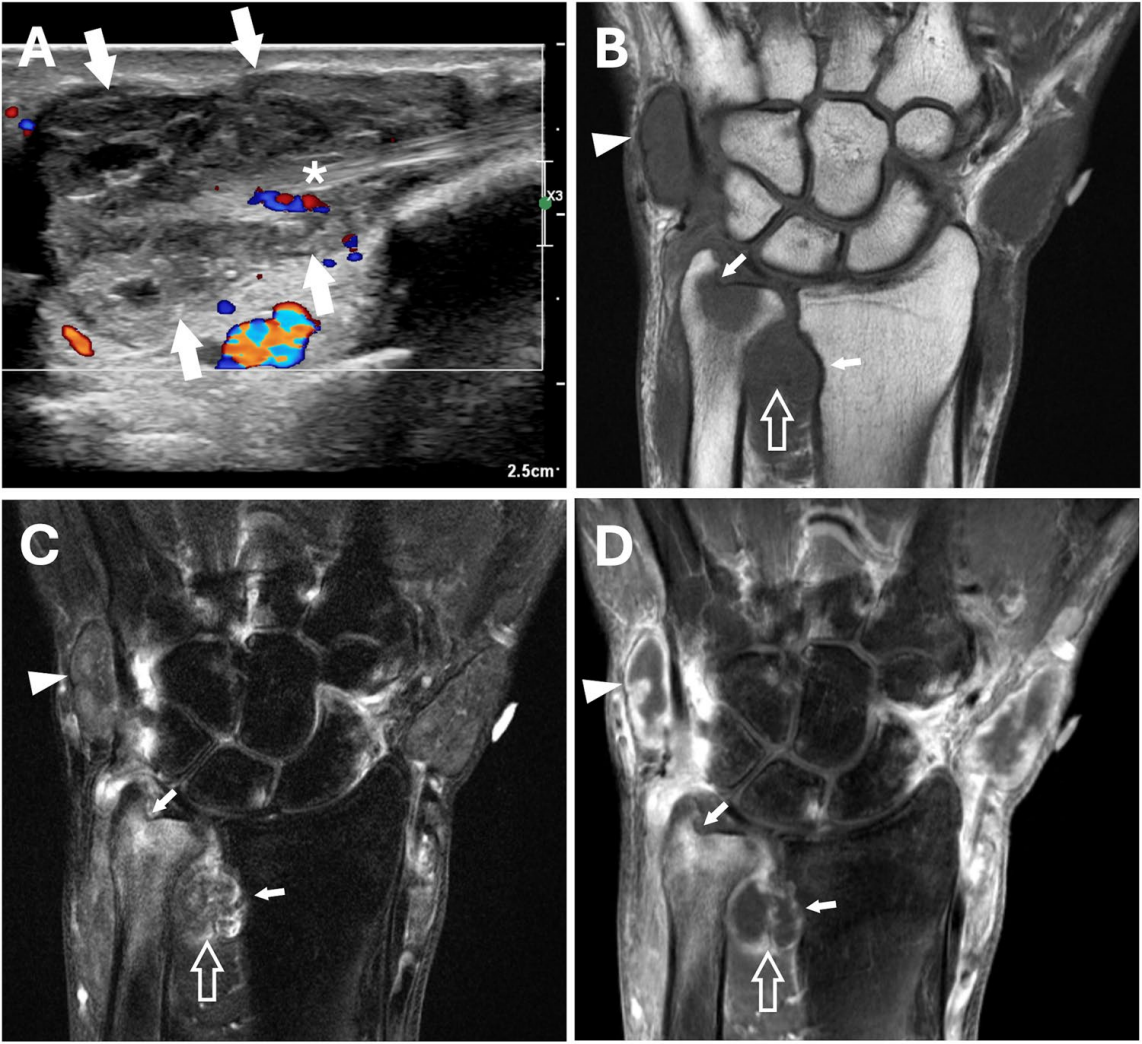
US and MR imaging of the right wrist. (**A**) Long axis image of the extensor pollicis brevis (EPB) tendon sheath at the level of the base of the first metacarpal shows a heterogeneous, predominantly hypoechoic and hypovascular mass (arrows) surrounding the tendon (asterisk). Coronal (**B**) T1-weighted, (**C**) T2-weighted fat-suppressed, and (**D**) post-contrast T1-weighted fat-suppressed MR images show marked distension of the EPB tendon sheath (external marker), extensor carpi ulnaris tendon sheath (arrowheads), and distal radioulnar joint (open arrows) with variably sized, relatively hypointense and hypoenhancing masses surrounded by synovitis. Scattered osseous erosions are present about the wrist, most notably at the sigmoid notch of the radius and ulnar fovea (small arrows), with variable edema-like signal and enhancement

**Fig. 2:**
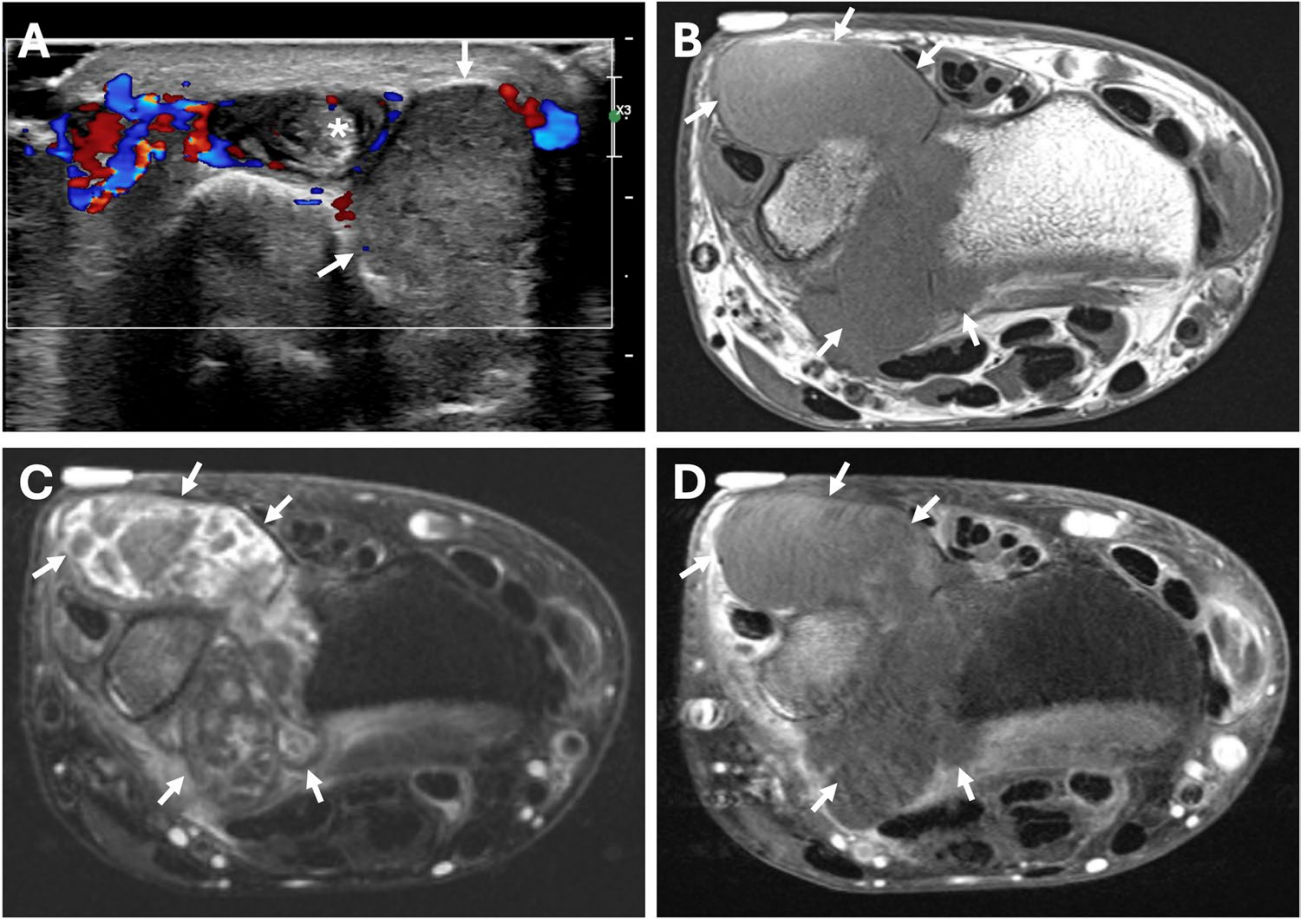
US and MR imaging of the right wrist. (**A**) Short axis image of the dorsal-ulnar aspect of the wrist shows iso-echoic, predominantly hypovascular mass-like distension of the dorsal recess of the distal radioulnar joint (arrows). There is also synovial proliferation within the extensor carpi ulnaris tendon sheath surrounding the tendon (asterisk). Axial (**B**) T1-weighted, (**C**) T2-weighted fat-suppressed, and (**D)** post-contrast T1-weighted fat-suppressed MR images show marked distension of the distal radioulnar joint (arrows) with variably sized, relatively hypointense and hypoenhancing masses and nodules

**Fig. 3:**
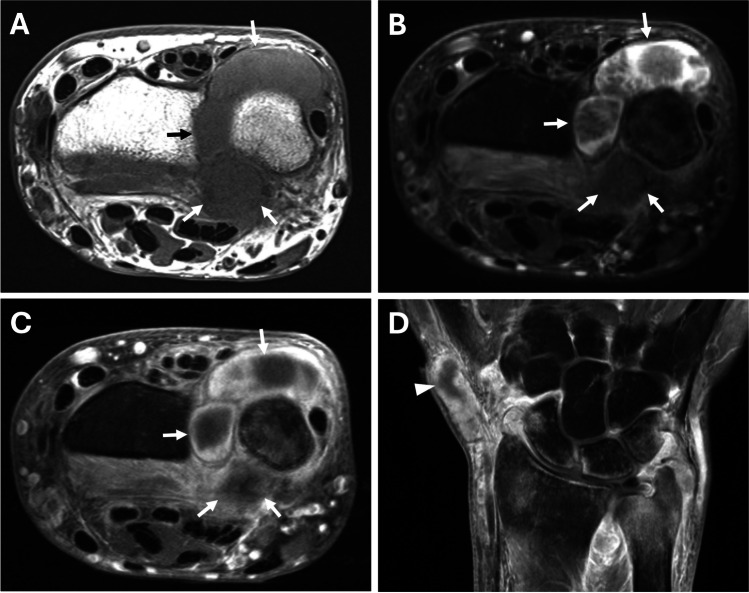
MR imaging of the left wrist. (**A**) Axial T1-weighted, (**B**) axial T2-weighted fat-suppressed, as well as (**C**) axial and (**D**) coronal post-contrast T1-weighted fat-suppressed MR images show relatively symmetric findings with the right wrist, including marked distension of the distal radioulnar joint (arrows) and extensor pollicis brevis tendon sheath (arrowhead) with variably sized, relatively hypointense and hypoenhancing masses and nodules. Polyostotic variable edema-like signal and enhancement is present throughout the wrist

Ultrasound (US) and magnetic resonance (MR) imaging was performed on both wrists (Figs. 1, 2, and 3). Imaging findings were relatively symmetric with marked distension of the distal radioulnar joints and extensor tendon sheaths. The compartments were filled with masses and nodules of various sizes surrounded by synovitis. On US imaging, the masses were heterogeneous but mostly hypo- to iso-echoic compared to muscle and relatively hypovascular. On MR imaging, compared to muscle, the nodules exhibited iso-intense signal on T1-weighted images, iso- to mildly hyper-intense signal on T2-weighted fat-suppressed images, and minimal enhancement on post-contrast images. Abundant enhancing synovitis surrounded the nodules with variably enhancing erosions at the sigmoid notch of the radius, distal ulna, and several carpal bones.

**Fig. 4:**
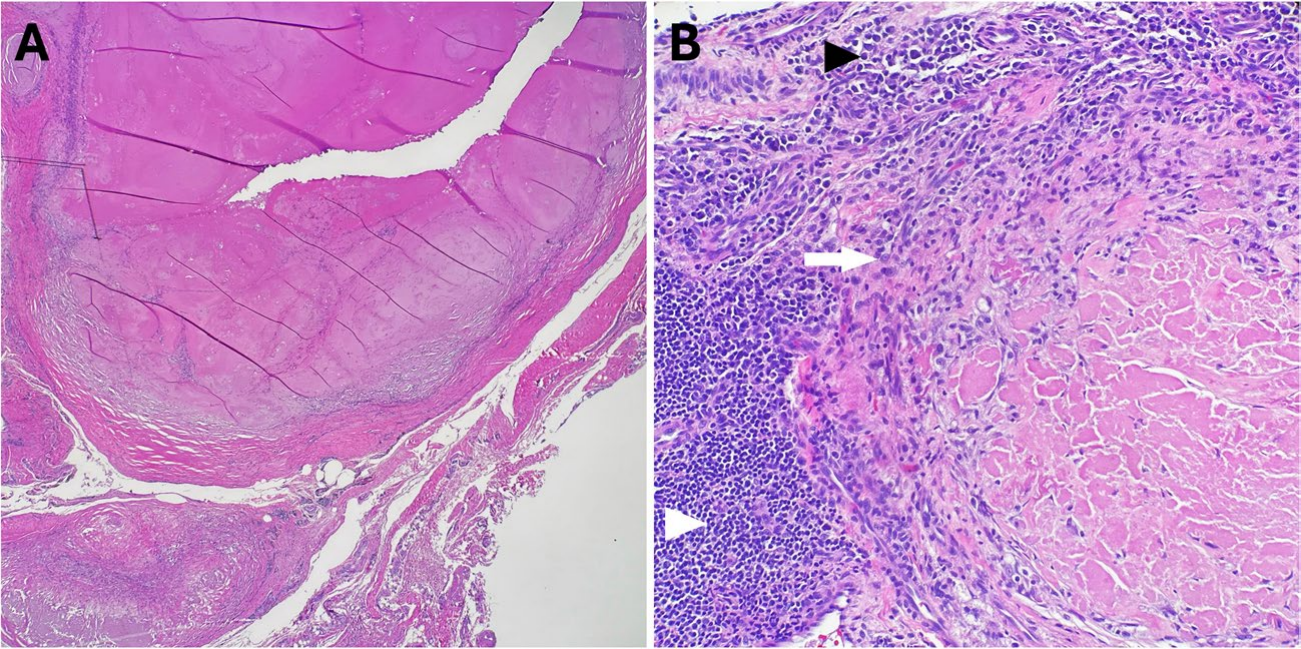
Representative histologic images. (**A**) Low-power magnification demonstrates large hyalinizing granulomas. (**B**) High-power magnification shows associated chronic inflammation, predominantly comprised of lymphocytes (white arrowhead), histiocytes (arrow), and plasma cells (black arrowhead)

Open surgical biopsy was performed at multiple sites in the right wrist. Tan-gray rubbery tissues were removed from the extensor pollicis brevis tendon sheath measuring 2.0 × 1.7 × 1.5 cm (corresponding to the imaging location in Fig. 1A) and expanded dorsal recess of the distal radioulnar joint measuring 2.5 × 2.0 × 0.7 cm (corresponding to the imaging location in Fig. 2A). Pathologic evaluation revealed synovium containing large, hyalinized granulomas, chronic inflammation, granulation-type tissue, hemosiderin deposition, and reactive changes (Fig. 4). No discrete collections of organizing fibrin were noted, nor was significant nuclear atypia or increased mitotic activity observed. Crystal evaluation was negative on polarized microscopy. No organisms were detected with Gomori methenamine silver, periodic acid-Schiff, and acid-fast bacillus stains. Congo red staining showed no amyloid deposition. Immunostaining with CD138 showed a few plasma cells which were polytypic for kappa and lambda light chains, CD68 which showed a few histiocytes, and pan-keratin which was negative.

The final diagnosis of hyalinizing granulomas was made through multidisciplinary consultation between rheumatology, musculoskeletal radiology, dermatopathology, hematopathology, and infectious disease pathology. In light of this unique diagnosis, the patient was started on a daily treatment of 4 mg of methylprednisolone. Following this, the patient experienced marked improvement in symptoms and a significant reduction in the size of the nodules. Notably, the nodules that were surgically removed for analysis showed no signs of regrowth.

## Discussion

PHG is a rare, benign lung disease typically occurring in middle age, affecting both genders with equal frequency [1, 4]. Extrapulmonary spread of PHG is exceedingly rare, with only a few reported cases with histologic confirmation, including involvement of the skin, tonsillar or subglottic area, pleura, pericardium, optic nerve, pituitary gland, liver, spleen, and abdomen [5, 6, 10]. Laraki et al. described two patients with PHG, including one with limited range of motion in the elbow, which the authors presumed to represent coexistent EPHG due to resolution of symptoms with corticosteroid therapy [4]. However, the only imaging performed of the elbow were radiographs which did not show any lesions and there was no pathological evaluation at that location. To date, there exists only a single published case of isolated EPHG (without pulmonary involvement), which occurred intracranially in the falx cerebri [11]. Thus, our current case represents the second published case of isolated EPHG and the first involving the musculoskeletal system.

The etiology of PHG remains poorly understood, with multiple authors having hypothesized that it represents an exaggerated chronic immune response to the antigenic stimuli associated with various infections and immune-mediated conditions affecting the lungs [1, 5, 7–9]. Additionally, associations with non-pulmonary pathological conditions have been observed, including idiopathic thrombocytopenic purpura, deep venous thrombosis, human immunodeficiency virus infection, and several autoimmune diseases such as Graves’ disease, sarcoidosis, cutaneous vasculitis, antiphospholipid syndrome, and multiple sclerosis, along with rheumatoid arthritis, Sjögren syndrome, autoimmune thyroiditis, IgA nephropathy, hyper-IgG4 syndrome, and ANCA-associated vasculitis [1, 6, 9, 12–15]. Reports also indicate that PHG can coexist with mediastinal and retroperitoneal fibrosis and fibrous lesions in other sites, such as the kidney, tonsil, and thyroid gland [3, 6, 9, 16]. Furthermore, there have been instances of PHG occurring alongside lymphoproliferative disorders, including lymphoma and Castleman’s disease [8]. In the context of this case, our patient’s history is particularly noteworthy with CLL and idiopathic thrombocytopenic purpura, conditions that align with the spectrum of disorders often associated with extrapulmonary manifestations. In the lungs, symptoms of PHG are mild and nonspecific and may include a cough, fever, fatigue, dyspnea, and pleuritic chest pain [9]. Symptomatic autoimmune diseases associated with PHG have included posterior uveitis, hemolytic anemia, anemia of chronic disease, amyloidosis, Sjogren’s syndrome, primary biliary cirrhosis, rheumatoid arthritis, and membranous glomerulonephritis [6, 8].

Definitive diagnosis is through histological examination, typically showing nodules which are composed of sclerotic, hyaline collagen bundles arranged in a haphazard manner. These bundles sometimes form concentric patterns around blood vessels. The lesions often exhibit mild infiltration of plasma cells, lymphocytes, and sporadic giant cells, particularly near the margins. Notably, there have been no reports of vasculitis or active infection in these lesions [4, 17]. In the case we are reporting, the open biopsy results showed hyalinizing granulomatous inflammation without evidence of infection or significant atypia. The absence of amyloid deposition, organisms, and atypical cells, along with the presence of polytypic plasma cells and histiocytes, is consistent with a non-infectious, inflammatory etiology. These findings are characteristic of hyalinized granulomas, and the lack of pulmonary findings align with isolated EPHG.

In terms of imaging characteristics, PHG typically manifests in the lungs as isolated or multiple nodular lesions, ranging from 1 mm to 10 cm in size, best characterized on computed tomography. These nodules sometimes present with excavation and/or calcification. Follow-up imaging often demonstrates slow growth and a tendency for these lesions to coalesce over time [6, 18]. 18F-FDG PET-CT scans reveal hypermetabolism in the nodules of 60% of patients with PHG [6, 9]. Regarding EPHG, among the documented cases, detailed imaging characteristics have only been provided for extrapulmonary localization to the pituitary gland [10]. In this case, a CT scan of the head showed a lesion involving the cavernous sinus and right orbital apex, invading the lateral wall of the sphenoid sinus. On MR imaging, the lesion demonstrated an isointense signal on T1-weighted images, a hypointense signal on T2-weighted images, and homogeneous enhancement after injection of gadolinium [10].

In the case we are reporting, US and MR imaging showed marked distension of the distal radioulnar joints and tendon sheaths. Distinct nodules of various sizes were noted, surrounded by synovitis. Amyloidosis was considered in the differential diagnosis as there are overlapping imaging appearances with our case, including nodules with relatively low signal intensity on both T1- and T2-weighted images, moderate enhancement, and erosions. Sarcoidosis can also present with variably sized nodules with intra-articular and tenosynovial involvement, but the nodules typically demonstrate hyper-intense signal on T2-weighted images with enhancement. Rice bodies were also considered, along with the associated differential diagnosis including infection or inflammation such rheumatoid arthritis, but the nodules encountered in our case were substantially larger. Furthermore, the histologic feature characteristics of rice bodies were lacking in our case; discrete collections of organizing fibrin were notably absent, as was the expected gross examination finding of numerous pale-tan and smooth loose bodies. A wide spectrum of imaging appearances can be seen with gout, and this was also included in the differential. Although clinical and laboratory results were helpful in narrowing the differential, ultimately the diagnosis of EPHG could only be made pathologically.

The natural course of PHG/EPHG is most often benign, typically with no recurrence observed following the excision of a solitary pulmonary nodule [4, 8]. While the efficacy of glucocorticoids in treating PHG/EPHG has not been conclusively established, responses in some cases suggest that these lesions might represent various manifestations of a systemic immunological disorder affecting multiple organs [3, 4, 6, 8]. In our specific case, after receiving a histopathological diagnosis, the patient was started on a daily treatment of 4 mg of methylprednisolone with rapid marked improvement in symptoms and a significant reduction in the size of the nodules. This particular response to steroid therapy, along with the absence of regrowth in excised nodules, is indicative of extrapulmonary hyalinizing granuloma, aligning with the observations in other cases.

In conclusion, isolated EPHG is exceedingly rare diagnosis and can involve the joint and tendon sheaths. The unique occurrence of EPHG, distinct from the more commonly recognized intrapulmonary manifestations, underscores the condition’s diverse clinical presentation. Although the specific etiology of EPHG remains largely unknown, its association with various autoimmune and hematologic conditions necessitates a thorough evaluation in affected patients. As demonstrated in our case, glucocorticoids may provide effective treatment, suggesting an underlying immunological component. Accurate diagnosis of EPHG hinges on histological examination, highlighting the importance of invasive diagnostic techniques. Each reported case of EPHG, including ours, significantly enriches the medical literature by shedding light on its clinical presentation, diagnosis, and management strategies. The generally favorable prognosis of EPHG is tempered by its potential association with systemic conditions, which should inform both treatment plans and prognostic evaluations. Therefore, the management of EPHG should adopt a holistic approach, addressing not only the local manifestations but also any concurrent systemic disorders. This case underscores the need for increased awareness and documentation of EPHG to enhance our understanding and improve patient care.
